# A data-driven approach for modelling Karst spring discharge using transfer function noise models

**DOI:** 10.1007/s12665-023-11012-z

**Published:** 2023-06-24

**Authors:** Max Gustav Rudolph, Raoul Alexander Collenteur, Alireza Kavousi, Markus Giese, Thomas Wöhling, Steffen Birk, Andreas Hartmann, Thomas Reimann

**Affiliations:** 1 Institute of Groundwater Management, Technische Universität Dresden, Dresden, Germany; 2 Department Water Resources and Drinking Water, Eawag, Dübendorf, Switzerland; 3 Department of Earth Sciences, University of Gothenburg, Gothenburg, Sweden; 4 Chair of Hydrology, Institute of Hydrology and Meteorology, Technische Universität Dresden, Dresden, Germany; 5 Institute of Earth Sciences, NAWI Graz Geocenter, University of Graz, Graz, Austria

**Keywords:** Modelling, Karst, Uncertainty quantification, Transfer functions, Spring discharge

## Abstract

Karst aquifers are important sources of fresh water on a global scale. The hydrological modelling of karst spring discharge, however, still poses a challenge. In this study we apply a transfer function noise (TFN) model in combination with a bucket-type recharge model to simulate karst spring discharge. The application of the noise model for the residual series has the advantage that it is more consistent with assumptions for optimization such as homoscedasticity and independence. In an earlier hydrological modeling study, named Karst Modeling Challenge (KMC; Jeannin et al., J Hydrol 600:126–508, 2021), several modelling approaches were compared for the Milandre Karst System in Switzerland. This serves as a benchmark and we apply the TFN model to KMC data, subsequently comparing the results to other models. Using different data-model-combinations, the most promising data-model-combination is identified in a three-step least-squares calibration. To quantify uncertainty, the Bayesian approach of Markov-chain Monte Carlo (MCMC) sampling is subsequently used with uniform priors for the previously identified best data-model combination. The MCMC maximum likelihood solution is used to simulate spring discharge for a previously unseen testing period, indicating a superior performance compared to all other models in the KMC. It is found that the model gives a physically feasible representation of the system, which is supported by field measurements. While the TFN model simulated rising limbs and flood recession especially well, medium and baseflow conditions were not represented as accurately. The TFN approach poses a well-performing data-driven alternative to other approaches that should be considered in future studies.

## Introduction

Karst aquifers contain important fresh water resources for about $10 \; \%$ of the world’s population (Stevanović 2018) and act as a major resource for ecosystems, economic activities, agriculture, tourism, and recreation (Bakalowicz 2005; Martos-Rosillo et al. 2015; Olarinoye et al. 2020). Their highly heterogeneous and hierarchically organized subsurface structure originates from small-scale primary porosity in the soluble rock matrix, secondary porosity of fissures and fractures, and the large-scale tertiary porosity in conduits and larger voids. This structure gives rise to particular groundwater flow phenomena (e.g., Bakalowicz 2005; Hartmann et al. 2014). These manifest themselves in dynamic dualities of recharge processes, groundwater flow fields, and (spring) discharge (Kiraly 1998; Hartmann et al. 2013). Due to these dualities, the presence of turbulent flow, and threshold effects, karst system discharge responds non-linearly to precipitation (Pinault et al. 2001b; Bakalowicz 2005; Cuchi et al. 2014). Generally, karst system hydraulic behaviour during recession may be characterized by an exponential or generalized power-law function (Maillet 1905; Dewandel et al. 2003; Hergarten and Birk 2007; Kovács and Sauter 2008; Birk and Hergarten 2010); the detailed modelling of karst systems is, however, complicated due to heterogeneity, data scarcity, and system non-linearity.

Various different methods were developed to model karst system hydraulics and karst system spring discharge. *Lumped models*, such as bucket-type or time series and transfer function models, assume that karst systems transform the input signal (e.g., precipitation) into an output signal (i.e., spring discharge) without explicitly representing the (spatially distributed) physical processes of this transformation. *Distributed models* represent the system in a spatially distributed way, enabling a process-based representation and insight into hydraulic parameter fields. Summaries and descriptions of the different methods can be found in, for example, Goldscheider and Drew (2014), Hartmann et al. (2014) and Jeannin et al. (2021).

To compare the performance of different modelling approaches for forecasting spring discharge, the Karst Modelling Challenge (KMC) (Jeannin et al. 2021) was initiated. There, 13 different models—from neural networks over bucket-type models to distributed models—were compared by calibrating all models on the same data sets (meteorological forcing data and spring discharge data). The different approaches were then compared on a previously unused and unknown testing period where only meteorological forcing data were given. Other than neural networks, no time series approaches were compared in the KMC.

The objective of this paper is to assess the suitability of time series models, specifically transfer function noise (TFN) models using impulse response functions in continuous time, for the simulation of karst spring discharge. Time series models —and transfer function approaches specifically—are lumped models and they have been shown in the past to perform well at modelling karst system spring discharge (e.g., Labat et al. 1999, 2000; Pinault et al. 2001a, c; Denić-Jukić and Jukić 2003; Jukić and Denić-Jukić 2004, 2006; Ladouche et al. 2014; Cuchi et al. 2014). Transfer function approaches are most commonly utilized to study linear time invariant (LTI) systems (e.g., Ljung 1999) but it is common practice to approximate non-linear systems, such as karst systems, with linear methods. This approximation is motivated by karst systems being time invariant over human time scales (Cuchi et al. 2014) and by the successful application of linear methods for karst systems (see references above). The transfer function noise modelling approach used in this study has been used in the past for modelling heads in unconsolidated aquifers (e.g., von Asmuth et al. 2002) but has never been tested for karst system spring discharge simulation. Furthermore, TFN modelling is coupled in this study with a bucket-type recharge model to account for the nonlinear response of recharge to precipitation (Peterson and Western 2014; Collenteur et al. 2021), which is an additional novelty in karst hydrological modelling.

A common notion in model calibration is to minimize the series of residuals between simulated outputs and observations. Many minimization and uncertainty quantification methods rely on the assumption that the residuals are independent and identically (normally) distributed (IID) (e.g., Nocedal and Wright 2006; Sullivan 2015; Ghanem et al. 2017). In hydrological modelling, however, residuals are often autocorrelated and not normally distributed (e.g., von Asmuth and Bierkens 2005; Evin et al. 2013, 2014). Nevertheless, minimization algorithms are still ubiquitously applied, despite the assumptions often not being met or checked. To make robust parameter inferences in the inverse problem, a noise model is employed on the residual series in this study. This simultaneously aims at reducing autocorrelation in the residual series and at meeting the assumption of IID residuals (von Asmuth et al. 2002; Vrugt 2016; Vrugt et al. 2009; Collenteur et al. 2019).

For solving inverse problems, i.e., model calibration, analyzing parameter and simulation uncertainties is vital for model performance assessment (Gupta et al. 2005; White et al. 2014, 2016; Teixeira Parente et al. 2019). Model structural or conceptual uncertainties were analyzed in the past for lumped (e.g., Hartmann et al. 2013; Hartmann 2018; Schuler et al. 2020) and distributed (Fandel et al. 2021) models for karst spring discharge. Model parameter uncertainty and the resulting simulation uncertainty, however, are rarely quantified (e.g., Teixeira Parente et al. 2019). A Bayesian framework of uncertainty quantification (UQ) is adopted in this study. The beliefs about the prior model parameter distributions are conditioned on observed data to obtain posterior parameter distributions, which are sampled via Markov-chain Monte Carlo (e.g., Sullivan 2015; Ghanem et al. 2017; Teixeira Parente et al. 2019). Noise is incorporated in the likelihood function for Bayesian inversion, better meeting the underlying assumptions of IID residuals (e.g., Vrugt 2016; Vrugt et al. 2009; Evin et al. 2013, 2014).

In this study, the applicability of TFN models using predefined response functions together with a non-linear bucket-type recharge model is tested to simulate karst spring discharge. The methodology of TFN model calibration and Bayesian UQ is applied to the KMC data, enabling the comparison of the obtained results to various other modelling approaches.

## Materials and methods

### Study site

**Fig. 1:**
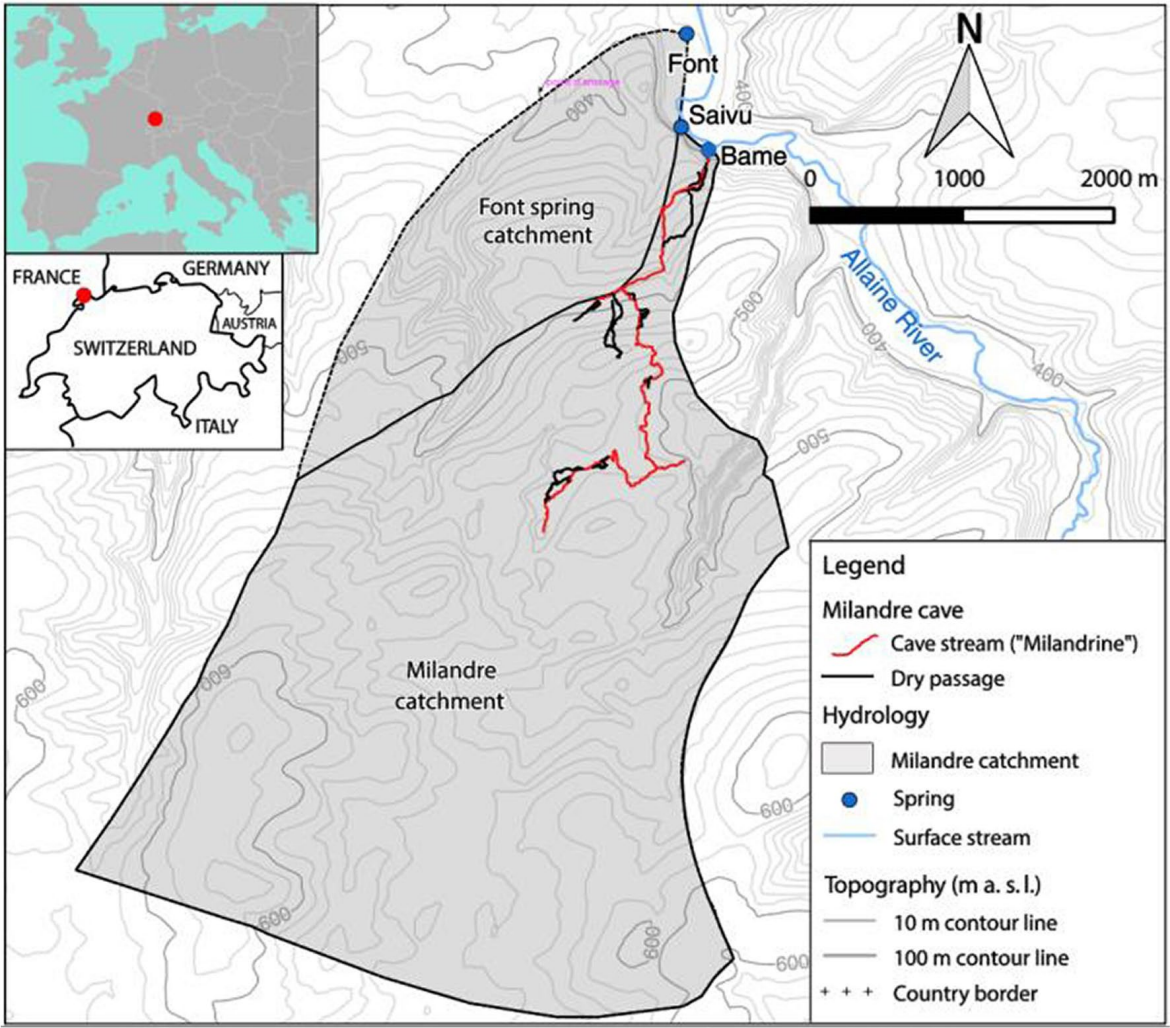
Map of the study site of the Milandre karst system catchment with its main characteristics (Jeannin et al. 2021)

The methods of TFN modelling and inversion are tested on the shallow and well-karstified Milandre Karst System (MKS), which has been intensively modelled and investigated in previous studies (Grasso and Jeannin 1994; Jeannin 1998; Kovács and Jeannin 2003; Perrin 2003; Perrin et al. 2003). The MKS is also the study area for the Karst Modeling Challenge (KMC) (Jeannin et al. 2021). We refer the reader to Jeannin et al. (2021) and references therein for a more detailed description of the case study area. The MKS is located in northern Switzerland on the north-western side of the Jura mountains. In Fig. 1, the study site as well as its main characteristics are presented.

The recharge area can be characterized as a limestone plateau, which is covered by forests, pastures and cultivated land, each at approximately $30\%$ of the total area, while approximately $5\%$ of the area is covered by urban regions. $95\%$ of the limestone area are covered by soil. The soil thickness ranges from 0.3 to 0.5 m in the forests, whereas in the cultivated land the thickness can be up to 2.0 m (Jeannin et al. 2021). Perrin et al. (2003) characterized the soil to consist mainly of cohesive silt loam. The recharge regime of the MKS is completely autogenic and diffuse, i.e., no swallow holes or surface streams are present. According to Perrin et al. (2003), the unsaturated zone has a thickness in the range of 40–80 m, whereas the saturated zone has a thickness of a few tens of meters.

### Available data

Jeannin et al. (2021) found the data of the Fahy meteorological station, located at about 7 km from the catchment center, to be most useful for the purpose of reliable simulation of spring discharges. Therefore, the analysis presented here was restricted to data from Fahy station. Time series of meteorological measurements were available from 1992-01-01 to 1995-12-31, from 2014-01-01 to 2015-12-31, and from 2016-01-01 to 2016-12-31 with an hourly frequency for precipitation and air temperature and daily frequency for evapotranspiration measurements. Total spring discharge, discharge water temperature, and electrical conductivity data were available from 1992-09-24 to 1995-03-28 and from 2014-01-01 to 2015-12-31 in hourly frequency. Data from 1992 to 1995 and from 2014 to 2015 could be used for calibration, data from 2016 could be used for testing (see “Least-squares optimization and noise modeling”). The data available in the present study is equivalent to the data available for participants in the KMC, enabling the comparison of results. During the testing period, no data was available for spring discharge, electrical conductivity, and discharge water temperature. Therefore, the model could not be evaluated by the authors of this study but evaluation of model results was performed by the main author of the KMC, P.-Y. Jeannin.

In this study, precipitation, evapotranspiration, and spring discharge data was used with hourly frequency. Therefore, daily evapotranspiration measurements were resampled to an hourly frequency through linear interpolation. All data that were used are shown in Fig. 9 and summarized in Table 1.

**Table 1:** Overview of available data with associated periods of availability

Precipitation	Evapotranspiration	Spring discharge	Use
1992-01-01–1995-12-31	1992-01-01–1995-12-31	1992-09-24–1995-03-28	Calibration
2014-01-01–2015-12-31	2014-01-01–2015-12-31	2014-01-01–2015-12-31	Calibration
2016-01-01–2016-12-31	2016-01-01–2016-12-31	–	Testing

### Transfer function noise model

#### Modeling framework

**Fig. 2:**
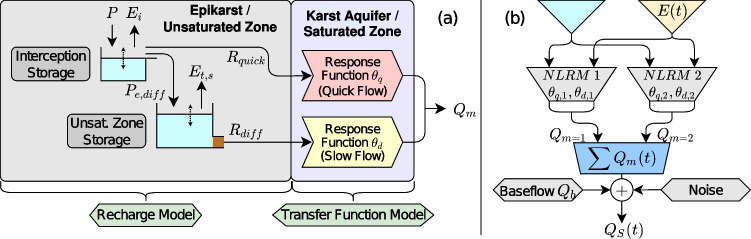
Recharge and TFN model structure; **a** non-linear two-component recharge model (NLRM), the root zone storage is implemented as a non-linear reservoir; **b** complete model structure, each NLRM in (**b**) represents (**a**); all symbols are explained within the text

In this study, the TFN model, proposed by von Asmuth et al. (2002) to simulate hydraulic heads, is adapted for the purpose of modelling spring discharge. The TFN model relates a system output time series (here, spring discharge) to an arbitrary number of system inputs or stresses (here, evapotranspiration and precipitation). Spring discharge $Q_S(t) \; [L^3 T^{-1}]$ is modelled as the sum of all *M* contributions $Q_m(t) \; [L^3 T^{-1}]$ from the different stresses *m* ($S_m(t) \; [LT^{-1}]$), a base flow component $Q_b \; [L^3 T^{-1}]$ and the residual series $r(t) \; [L^3 T^{-1}]$ (Eq. (1)). It is noted that the dimensions of the terms or variables may change, depending on the context of the problem studied. Residuals *r*(*t*) are calculated by subtracting simulated values from observed values. Furthermore, stresses may be used multiple times to compute different contributions as shown in Fig. 2.

1 $$\begin{aligned} Q_S(t) = \sum _{m=1}^{M} Q_m(t) + Q_b + r(t). \end{aligned}$$

The contribution $Q_m(t)$ is calculated by the convolution of a stress time series with an impulse response function (von Asmuth et al. 2002), given in discrete time as

2 $$\begin{aligned} Q_m(t) = \sum _{\tau = 0}^{t} S_m(\tau ) \theta _m(t - \tau ), \end{aligned}$$

where $\theta _m(t)$ is the impulse response function describing how the system reacts to an infinitely short pulse of stress $S_m$.

A step response $\Theta (t)$ can be obtained by integrating the impulse response function over time. The step response represents the system response due to a constant stress (e.g., continuous precipitation at a unit rate) starting at $t = 0$.

3 $$\begin{aligned} \Theta (t) = \int _0^t \theta _m(t) \textrm{d}t. \end{aligned}$$

The step response eventually converges to a steady state response as $t \rightarrow \infty$, giving the maximum gain of the response. The gain may be interpreted here as the resulting spring discharge contribution if it precipitates at unit rate infinitely in time. The response function represents the unit response of certain reservoirs or combinations of those (Denić-Jukić and Jukić 2003).

#### Recharge representation

The karst system recharge process has to be represented using multiple response functions due to the corresponding duality (direct and indirect/diffuse recharge) (Pinault et al. 2001c, a; Ladouche et al. 2014). To make hydraulic heads non-linearly related to precipitation and potential evaporation, Peterson and Western (2014) proposed and tested the use of a soil–water balance approach for the calculation of recharge. Collenteur et al. (2021) tested this approach for hydraulic heads in Austria and obtained good results. The bucket-type non-linear recharge model developed in Collenteur et al. (2021) is extended in this study to represent the two flow components of recharge, i.e., the duality of the recharge process in karst aquifers. One response function is subsequently applied for each flow component, as done in previous studies (Pinault et al. 2001c, a; Ladouche et al. 2014). A schematic representation of the extended recharge model is presented in Fig. 2a.

A detailed description of the recharge model can be found in Collenteur et al. (2021), an explanation is given for the recharge model adaption here. After the maximum capacity of the interception storage $S_{i, \max } \; [L]$ is reached, additional precipitation overflows as effective precipitation $P_e \; [LT^{-1}]$. Instead of routing $P_e$ entirely to the root zone storage as in the original formulation, $P_e$ is divided into a quick recharge component $R_{\text {quick}} \; [LT^{-1}]$ (see Eq. (10)) and a diffuse effective precipitation component $P_{e, \text {diff}} \; [LT^{-1}]$ (see Eq. (7)). The latter is routed to the root zone storage and the division or fractionation is controlled by the factor $\omega _f \; [-]$. The interception storage reservoir of the recharge model is governed by the following equations:

4 $$\begin{aligned} \frac{\Delta S_i}{\Delta t}&= P - E_i - P_e , \end{aligned}$$

5 $$\begin{aligned} E_i \Delta t&= \textrm{min}(E_{\max } \Delta t, S_i) , \end{aligned}$$

6 $$\begin{aligned} P_e&= {\left\{ \begin{array}{ll} S_i - S_{i, \max } &{}\text {if } S_i > S_{i, \max } \\ 0 &{}\text {if } S_i \le S_{i, \max } \end{array}\right. }, \end{aligned}$$

where $S_i \; [L]$ is the current water level in the interception storage, $P \; [LT^{-1}]$ is the precipitation flux, $E_i \; [LT^{-1}]$ is the interception evaporation flux, $E_{\max } = k_v E_p, \; [LT^{-1}]$ is the vegetation-corrected evaporation flux with $k_v \; [-]$ being the vegetation factor and $E_p \; [LT^{-1}]$ being the potential evaporation flux. The root zone storage reservoir of the recharge model is governed by the following equations:

7 $$\begin{aligned} \frac{\textrm{d} S_r}{\textrm{d} t}&= (P_e (1 - \omega _f)) - E_{t, s} - R, \end{aligned}$$

8 $$\begin{aligned} E_{t, s}&= (E_{\max } - E_i) \textrm{min} \left[ 1, \frac{S_r}{l_p S_r} \right] \end{aligned}$$

9 $$\begin{aligned} R_{\text {diff}}&= K_s \left( \frac{S_r}{S_{r, \max }} \right) ^{\gamma } \end{aligned}$$

10 $$\begin{aligned} R_{\text {quick}}&= \omega _f P_e, \end{aligned}$$

where $S_r \; [L]$ is the water level in the root zone storage, $S_{r, \max } \; [L]$ is the storage capacity of the root zone storage reservoir, $l_p \; [-]$ gives the fraction of $S_{r, max}$ at which the soil evaporation is limited, $K_s \; [LT^{-1}]$ is the saturated hydraulic conductivity of the soil, $\gamma$ is the exponent controlling the flow non-linearity, and $R_{\text {diff}} \; [LT^{-1}]$ is the slow or diffuse recharge component.

Initially, at $t = 0$, the saturation in the root zone storage is set to $S_r (t=0) = 0.5 \; S_{r, \max }$ and the interception storage is empty ($S_i (t=0) = 0$). After the calculation of the recharge fluxes, each recharge flux gets convoluted according to Eq. (2) with the corresponding response function for the quick or diffuse flow component, respectively.

#### Response functions

Predefined impulse response functions are used to transform the computed recharge components (fast and slow flow) from the recharge model to the final discharge contributions (see also Eq. (2)). As such, the response functions account for the effect of the porous medium. Predefined response functions have the advantage of a smaller number of parameters compared to models using discrete non-parametric response functions (von Asmuth et al. 2002; Neuman and De Marsily 1976; Dreiss 1989). Using response functions in continuous time allows to calculate the system response at arbitrary time steps (von Asmuth et al. 2002). These aspects are also part of the well-established general theory on system identification and the reader is referred to Ljung (1999) for more information.

**Fig. 3:**
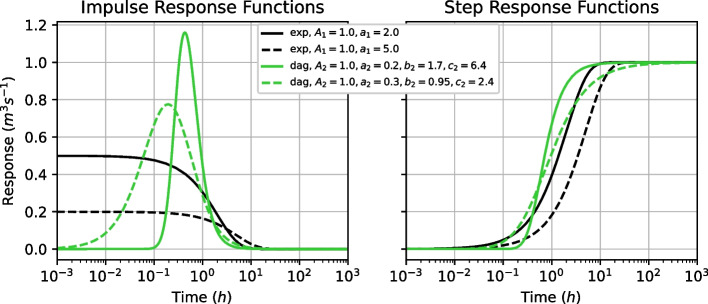
Response functions used with plausible shapes; **a** impulse response functions, **b** step response functions; response functions are abbreviated for Dagum (dag) and exponential (exp)

While the medium and late recession behaviour of karst spring discharge can be represented by an exponential function with a corresponding recession coefficient (Maillet 1905; Kovács and Sauter 2008), the early recession behaviour may be better represented following a power-law function (Hergarten and Birk 2007; Birk and Hergarten 2010). Following this, we use a scaled exponential function (Eqs. (11), (13)) and a scaled Dagum probability density function (Kleiber 2008) (Eqs. (12), (14)), which follows a power-law, as impulse response functions. The response functions are shown with typical parameter values in Fig. 3.

11 $$\begin{aligned} \theta _{\text {exp}}(t)&= \frac{A_1}{a_1} e^{-t/a_1}, \end{aligned}$$

12 $$\begin{aligned} \theta _{\text {dag}}(t)&= A_2 \frac{b_2c_2}{t} \left( \frac{\left( \frac{t}{a_2} \right) ^{b_2c_2}}{\left( \left( \frac{t}{a_2} \right) ^{b_2} + 1 \right) ^{c_2+1}} \right) , \end{aligned}$$

where $A_1 \; [L^3T^{-1}]$ and $A_2 \; [L^3]$ are the scaling parameters and $a_1 \; [T]$, $a_2 \; [T]$, $b_2 \; [-]$, and $c_2 \; [-]$ are shape parameters.

In the modelling approach used here, the step responses are used for the computations, which are given in Eqs. (13) and (14).

13 $$\begin{aligned} \Theta _{\text {exp}}(t)&= \int _0^{t} \theta _{\text {exp}}(t) \textrm{d}t = A_1 (1 - e^{-t / a_1}) , \end{aligned}$$

14 $$\begin{aligned} \Theta _{\text {dag}}(t)&= \int _0^{t} \theta _{\text {dag}}(t) \textrm{d}t = A_2 \left( 1 + \left( \frac{t}{a_2} \right) ^{-b_2} \right) ^{-c_2}. \end{aligned}$$

The physical interpretability of Eq. (13) is given by the relation of $a_1$ to the recession coefficient of the system (Maillet 1905; Kovács and Sauter 2008). Eq. (14) can be physically interpreted as well, where $A_2$ controls the overall scale of the response, $a_2$ controls the overall length of the response, $b_2$ controls the lag time of the response and $c_2$ is a damping parameter. However, no analogy to a certain reservoir type can be given as in Denić-Jukić and Jukić (2003); Ladouche et al. (2014).

All previously mentioned models and methods were implemented in an adapted version of the time series analysis software Pastas (Collenteur et al. 2019), based on version v0.16.0 (Collenteur et al. 2020). The code and scripts are available as supplementary materials (see Supplementary information).

#### Model structure

To represent the karst system conceptually, we employ an approach similar to Denić-Jukić and Jukić (2003), representing the response to diffuse and direct recharge individually. To account for conceptual or structural variability of the model, we employ two parallel recharge models (see Fig. 2) with two response functions for each recharge model, where one recharge model gets attributed with two exponential response functions (one for direct, one for diffuse discharge behaviour) and the other recharge model gets attributed with two Dagum response functions (one for direct, one for diffuse discharge behaviour).

Because all response functions feature a scale parameter ($A_1, A_2$ in Eqs. (11), (12)), the overall contribution of processes related to exponential or power-law behaviour is variable. Furthermore, the contribution of diffuse or direct system behaviour is correspondingly variable as well, enabling the representation of complex recession behaviour that dynamically changes from a power-law to an exponential. Therefore, although the structure of the recharge model is fixed, the parallel setup together with variable representation of system behaviour allows for a high degree of flexibility. The model structure is shown in Fig. 2b.

### Solving the inverse problem

#### General methodology

**Fig. 4:**
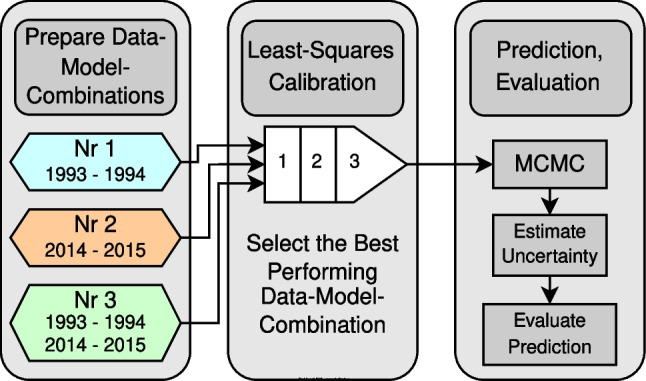
Inverse problem solution strategy; MCMC stands for Markov-chain Monte Carlo sampling as a method of Bayesian inversion; the numbers in the box for least-squares calibration represent the three steps described in “General methodology”, which are carried out with each data-model-combination

Ultimately, the goal of inversion in this study is to obtain the most probable simulations during calibration and testing periods together with associated uncertainty bounds. This can be achieved via Bayesian inversion, which is, however, computationally intensive. Multiple calibration data sets are available for the studied case, and thus different data-model-combinations exist. It is of interest (especially for practical applications) to avoid the computationally costly Bayesian inversion for all data-model-combinations. This is achieved by performing less computationally costly least-squares calibration first for all data-model-combinations and selecting the most promising data-model-combination afterwards. Then, Bayesian inversion is only performed for this most promising data-model-combination (see “Bayesian inversion and uncertainty quantification”). This general workflow is shown in Fig. 4.

Three different data-model-combinations are tested during least-squares calibration, using identical model structures (see also Fig. 9): for data-model-combination 1, system input and output data is used from 1993-01-01 00:00:00 to 1994-12-31 23:00:00; for data-model-combination 2, system input and output data from 2014-01-01 00:00:00 to 2015–12-31 23:00:00 is used; data-model-combination 3 considers both previously mentioned data sets. In the remainder, these three different data sets are termed calibration data.

Because the water levels in the reservoirs of the recharge models are initialized at the beginning of the simulation to an arbitrary level, a warmup period is used. Three full cycles of calibration data are prepended to the calibration period as a warmup period. Subsequently, a warmup period of 3650 days is used for all models during calibration. For data-model-combination 1 and data-model-combination 2, mean values are used to fill the remaining beginning of the warmup period.

For convenience, all spring discharge data units are transformed as $(m^3 s^{-1}) \rightarrow (10^3 \; m^3 s^{-1})$ before calibration. The total number of calibration parameters is $\textbf{m}=28$ for all data-model-combinations ($2 \times 7$ recharge model parameters, $2 \times 4$ parameters for the Dagum response function, $2 \times 2$ parameters for the exponential response function, 1 base flow parameter, 1 noise model parameter; see Table 3).

Previous studies with the Pastas package and the examples from the package documentation (Collenteur et al. 2020) suggest that it may have aggravating effects to simultaneously optimize all model parameters at once, starting from initial values, or to optimize model parameters together with the noise model parameter. Therefore, the least-squares calibration is divided into three consecutive steps. First, a run without a noise model is performed, having the parameters $\omega _f = 0.1, S_{r, \max } = 250.0 \; \text {mm}, l_p = 0.25, S_{i, \max } = 2.0 \; \text {mm}, k_v = 1.0$ fixed in both parallel recharge models. This is motivated by substantial correlation between the parameters and the corresponding aggravating effects of non-uniqueness and equifinality (Beven 2006; Gupta et al. 2009). In the second step, the calibration is continued reusing the optimized parameter set from the first step but varying all model parameters, still without a noise model. In the third and final step, the results from step 2 are used again together with a noise model, where the noise model parameter is optimized.

Afterwards, the calibration results from the third step of least-squares calibration are evaluated according to fit metrics for the calibration period for all data-model-combinations. Bayesian inversion (see “Bayesian inversion and uncertainty quantification”) is then performed with the most promising data-model-combination (see “Model fit metrics”) to obtain final simulations for the calibration and testing periods. Testing period performance for 2016 is finally assessed by the main author of KMC (P.-Y. Jeannin) and given in this study (Jeannin et al. 2021).

#### Least-squares optimization and noise modeling

The sum of the squared residual series from Eq. (1), *r*(*t*) , is minimized during least-squares optimization, obtaining a set of calibrated parameters. A noise model can be employed on *r*(*t*) to better meet statistical assumptions for parameter inference and uncertainty quantification (von Asmuth et al. 2002; von Asmuth and Bierkens 2005; Nocedal and Wright 2006; Vrugt 2016; Vrugt et al. 2009; Evin et al. 2013, 2014; Sullivan 2015; Ghanem et al. 2017). The equation for the autoregressive lag-1 (AR1) noise modelled residual series can be written as (e.g., von Asmuth et al. 2002)

15 $$\begin{aligned} \nu (t) = r(t) - e^{- \Delta t / \alpha } \; r(t - \Delta t), \end{aligned}$$

where $\nu (t)$ is the noise or future prediction error, i.e., the effect of random noise on the residuals between the time steps $t - \Delta t$ and *t*. There, $\Delta t$ is the time step length and $\alpha$ is a decay constant of the effect, which has to be calibrated.

To optimize the model parameters, the residual or noise series (see “General methodology”) is then minimized (Eqs. (16), (17))

16 $$\begin{aligned} \textbf{x}_{\text {opt, res}}&= \min _{\textbf{x} \in \mathcal {X}}{\{ r(\textbf{x}, t)^2: b_l \le \textbf{x} \le b_u \}}, \end{aligned}$$

17 $$\begin{aligned} \textbf{x}_{\text {opt, noise}}&= \min _{\textbf{x} \in \mathcal {X}}{\{ \nu (\textbf{x}, t)^2: b_l \le \textbf{x} \le b_u \}}, \end{aligned}$$

where $\textbf{x} \in \mathcal {X} \subseteq \textrm{R}^{\textbf{m}}$ is the vector of model parameters where the subscripts *opt*, *res*, and *noise* denote the optimal solution, residuals, and noise, respectively. $\textbf{m}$ is the number of model parameters and $(b_l, b_u)$ are the vectors of lower and upper bounds of the parameters (see Table 3), respectively. A non-linear least-squares algorithm, as implemented in the Python library Scipy (version 1.9.0) (Virtanen et al. 2020), is used to minimize the respective series.

#### Model fit metrics

Four fit metrics are used to evaluate the model performance during the calibration period for each data-model-combination according to criteria suggested in the KMC (Jeannin et al. 2021). The volume conservation criterion ($\overline{VCC}$, see Eq. (20) in the Appendix) is the ratio of the simulated flow volume and the observed one, where a value of 1 is the optimal outcome. The Nash-Sutcliffe efficiency (*NSE*, see Eq. (21) in the Appendix) incorporates the mean squared error as well as the variance and ranges between $- \infty < NSE \le 1$, where a value higher than $\approx 0.75$ indicates a reliable model (Nash and Sutcliffe 1970; Jeannin et al. 2021). The linearized version of the *NSE* ($NSE_{lin}$, see Eq. (22) in the Appendix) is used as well, which is not as sensitive to outliers as the original *NSE* (Legates and McCabe Jr. 1999). The Kling-Gupta efficiency (*KGE*, see Eq. (23) in the Appendix) is related to *NSE* but normalized to the range $0 \le KGE \le 1$ (Gupta et al. 2009). After the three-step least-squares calibration described in “General methodology”, the best performing data-model-combination is selected according to the combined $\overline{VCC}, NSE, NSE_{\text {lin}} \& \; KGE$ criteria and considered for Bayesian inversion.

#### Bayesian inversion and uncertainty quantification

Contrary to the deterministic approach of finding a single optimal parameter set with least-squares calibration, Bayesian inversion is a statistical approach based on Bayes’ theorem (Eq. (18)). There, the prior parameter distribution $p(\textbf{x})$ describes the belief about model parameters before taking any observed data, here $\textbf{D} = \{ Q_{S, t} \}_{t=0}^N$, into account. $p(\textbf{x})$ is then conditioned on observed data via the likelihood function $p(\textbf{D} \mid \textbf{x})$, which represents the probability of the model parameters when comparing the corresponding model simulation with observed data. This conditioning results in the posterior parameter distribution $p(\textbf{x} \mid \textbf{D})$. Therefore, parameter uncertainty can be directly quantified and model structural uncertainty is indirectly accounted for by using two parallel model parts (see “Model structure”). The present noise model furthermore indirectly introduces effects of data uncertainty (Vrugt 2016; Vrugt et al. 2009).

18 $$\begin{aligned} p(\textbf{x} \mid \textbf{D}) = \frac{p(\textbf{D} \mid \textbf{x}) p(\textbf{x})}{p(\textbf{D})} \propto p(\textbf{D} \mid \textbf{x}) p(\textbf{x}). \end{aligned}$$

Markov-chain Monte Carlo (MCMC) sampling is a highly popular method to sample from the (unnormalized) posterior density (e.g., Vrugt 2016; Vrugt et al. 2009; Teixeira Parente et al. 2019; Sullivan 2015), which is also used in this study. The general mechanism of MCMC is that a Markov-chain is generated in the parameter space (characterized by the prior distribution) by successively generating or proposing a new state (or sample) based on the previous state of the chain. A proposed state is then either accepted (resulting in a posterior sample) or rejected (resulting in a new proposal being made). Numerous MCMC algorithms are available; Pastas uses the emcee package (Foreman-Mackey et al. 2013) as given by the lmfit wrapper package (Newville et al. 2020). A parallelized affine-invariant ensemble sampler (AIES) algorithm is used (Goodman and Weare 2010), which runs multiple chains simultaneously. A uniform prior $p(\textbf{x})$ is assumed and the parameter prior ranges are given in Table 3. It is also assumed that the residuals are independent and follow a normal distribution, leading to the log-likelihood function $\textrm{ln} \mathcal {L}(\textbf{x}, \textbf{D}) = \textrm{ln}p(\textbf{D} \mid \textbf{x})$:

19 $$\begin{aligned} \textrm{ln} \mathcal {L}(\textbf{x}, \textbf{D}) = - \frac{1}{2} \sum _{t=0}^N \left( \frac{(\nu (\textbf{x}, t))^2}{\sigma _t^2} + \textrm{ln}(2 \pi \sigma _t^2) \right) , \end{aligned}$$

where $\sigma _t^2 \; [L^6T^{-2}]$ is the data variance. Using the noise series in place of the residuals ensures that the effect of noise is incorporated in Bayesian inversion. Therefore, the assumption of independent and identically (normally) distributed residuals can be met more accurately when using the noise series $\nu (\textbf{x}, t)$ compared to using the residual series $r(\textbf{x}, t)$ (Vrugt 2016; Vrugt et al. 2009; Evin et al. 2013, 2014).

All 28 parameters are varied using 100 walkers (chains), 12,000 steps, a burn-in period of 4000 steps together with a thinning of 4. MCMC sampling thus produces an array of samples with dimensions $[\mathrm {n_{samples}} \times \mathrm {n_{walkers}} \times \mathrm {n_{parameters}}] = [2000 \times 100 \times 28]$, giving a total of $2 \cdot 10^5$ samples from the parameter space. A final model simulation is then performed for the testing period using the parameter maximum likelihood estimates (MLE) from the MCMC sampling.

## Results

### Least-squares calibration

**Fig. 5:**
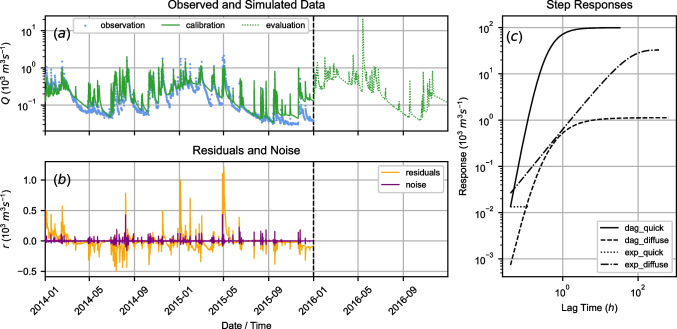
Results for data-model-combination 2 (utilizing data from 2014–2015) after least-squares calibration; response functions are abbreviated for Dagum (dag) and exponential (exp); **a** observed and simulated spring discharge, the vertical dashed line separates calibration and testing period; **b** residuals and noise; **c** calibrated step response functions

For an initial assessment of model performance of the three data-model-combinations, results are summarized in Table 2. More detailed results for data-model-combination 2 are given in Fig. 5. Detailed results for data-model-combinations 1 and 3 can be found in the Appendix in Figs. 10 and 11.

**Table 2:** Calibration fit metrics for all data-model-combinations (DMCs)

DMC	$\overline{VCC} \; [-]$	$NSE \; [-]$	$NSE_{lin} \; [-]$	$KGE \; [-]$
DMC1 (1993–1994)	3.050	0.206	– 0.043	0.631
DMC2 (2014–2015)	1.388	0.543	0.445	0.736
DMC3 (1994–1995 & 2014–2015)	1.694	0.449	0.323	0.680
MCMC (DMC2, 2014–2015)	1.188	0.598	0.512	0.779

From Figs. 5, 10, and 11 and the $\overline{VCC}$-values $> 1$ in Table 2 it was evident that flow was generally simulated too high, especially for moderate and base flow conditions. Peak flows were generally underestimated in data-model-combinations 1 and 3, whereas in data-model-combination 2 peak flows were represented more accurately. Even though a warmup period was used, the first $\approx 5$ months in the calibration periods were not simulated well; data-model-combination 1 resulted in flows being too high, data-model-combinations 2 and 3 resulted in flows being too low.

Taking the model calibration fit metrics in Table 2 into account, data-model-combination 2 was selected for further analysis (see “Model fit metrics”).

### Bayesian model calibration and uncertainty quantification

**Fig. 6:**
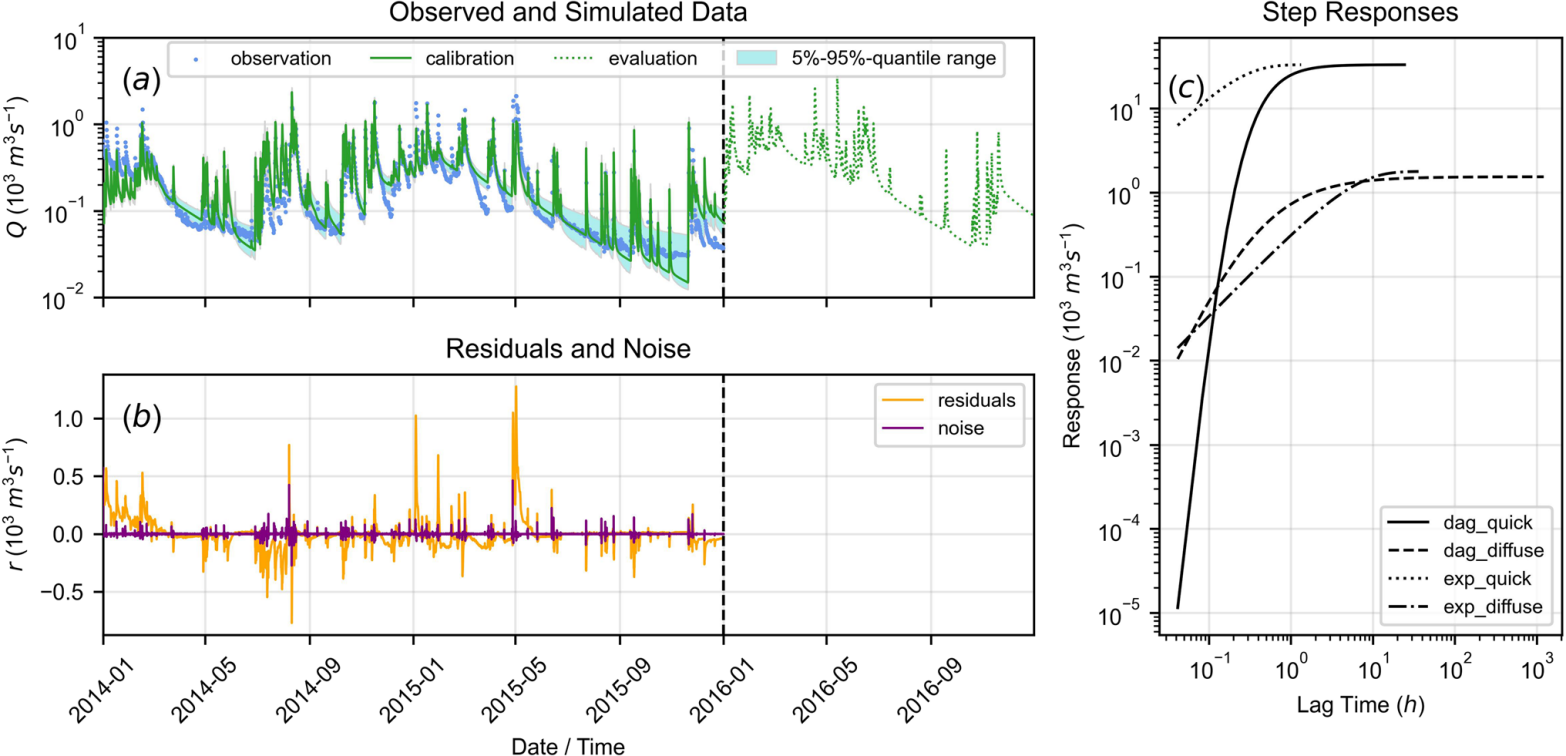
Results for data-model-combination 2 (utilizing data from 2014–2015) after Bayesian inversion with $5-95$%-quantile range obtained from 1000 randomly selected posterior samples; response functions are abbreviated for Dagum (dag) and exponential (exp); **a** observed and simulated spring discharge, the vertical dashed line separates calibration and testing period; **b** residuals and noise; **c** calibrated step response functions

For the MCMC sampling with multiple chains, the mean acceptance fraction was $\approx 10\%$. The maximum likelihood estimates (MLE) and the standard errors of the parameters are shown in Table 3. Results using the parameter MLE are shown in Fig. 6. Simulation uncertainty and median simulated flow are shown in Fig. 12.

**Table 3:** Inferred parameter values from Bayesian inversion with MCMC sampling; the second subscript of response function parameters denotes either quick (*q*) or diffuse (*d*) behaviour, the last subscript always denotes the corresponding recharge model number (i.e., 1 or 2); the standard error is given with respect to the MLE; abbreviations: Dagum response function (Dag.), exponential response function (exp.), root zone / interception storage (RS / IS), diffuse (diff.), limitation (lim.), saturated hydraulic conductivity (SHC), fractionation (fract.)

Parameter	Description	Units	Prior range	MLE value	Standard error
$A_{2, q, 1}$	Scale (quick Dag.)	$[10^3 \, m^3s^{-1}]$	[0.00, 100.00]	33.2	$19.0 \; (57.2 \%)$
$a_{2, q, 1}$	Shape (quick Dag.)	[*s*]	$[10^{-5}, 5 \cdot 10^3]$	0.20	$0.11 \; (55.1 \%)$
$b_{2, q, 1}$	Shape (quick Dag.)	$[-]$	$[10^{-5}, 5 \cdot 10^3]$	1.78	$0.59 \; (33.2 \%)$
$c_{2, q, 1}$	Shape (quick Dag.)	$[-]$	$[10^{-5}, 5 \cdot 10^3]$	5.25	$3.67 \; (70.0 \%)$
$A_{2, d, 1}$	Scale (diff. Dag.)	$[10^3 \, m^3s^{-1}]$	[0.00, 100.00]	1.54	$0.27 \; (17.7 \%)$
$a_{2, d, 1}$	Shape (diff. Dag.)	[s]	$[10^{-5}, 5 \cdot 10^3]$	0.38	$0.25 \; (65.4 \%)$
$b_{2, d, 1}$	Shape (diff. Dag.)	$[-]$	$[10^{-5}, 5 \cdot 10^3]$	0.96	$0.33 \; (34.5 \%)$
$c_{2, d, 1}$	Shape (diff. Dag.)	$[-]$	$[10^{-5}, 5 \cdot 10^3]$	2.23	$1.74 \; (77.9 \%)$
$S_{r, \max , 1}$	RS height	[mm]	$[10^{-5}, 10^4]$	145.2	$36.0 \; (24.8 \%)$
$l_{p, 1}$	RS evap. lim	$[-]$	$[10^{-5}, 1.00]$	0.02	$0.01 \; (68.8 \%)$
$K_{s, 1}$	SHC	[mm h$^{-1}$]	$[1.00, 10^4]$	2686.6	$1462.5 \; (54.4 \%)$
$\gamma _1$	Flow exponent	$[-]$	$[10^{-5}, 50.0]$	36.4	$12.4 \; (34.1 \%)$
$S_{i, \max , 1}$	IS height	[mm]	$[10^{-5}, 10.00]$	9.04	$1.53 \; (17.0 \%)$
$k_{v, 1}$	Evap. factor	$[-]$	$[10^{-6}, 10^2]$	0.88	$0.07 \; (7.53 \%)$
$\omega _{f, 1}$	Recharge fract	$[-]$	[0.00, 1.00]	0.016	$0.01 \; (62.5 \%)$
$A_{1, q, 2}$	Scale (quick exp.)	$[10^3 \, m^3 s^{-1}]$	$[10^{-5}, 2074.76]$	1.42	$6.55 \; (461.2 \%)$
$a_{1, q, 2}$	Shape (quick exp.)	$[-]$	$[0.01, 10^3]$	5.62	$7.56 \; (134.5 \%)$
$A_{1, d, 2}$	Scale (diff. exp.)	$[10^3 \, m^3 s^{-1}]$	$[10^{-5}, 1005.76]$	4.91	$0.91 \; (18.4 \%)$
$a_{1, d, 2}$	Shape (diff. exp.)	[*s*]	$[0.01, 10^3]$	69.8	$16.6 \; (23.8 \%)$
$S_{r, \max , 2}$	RS height	[mm]	$[10^{-5}, 5 \cdot 10^4]$	847.5	$280.5 \; (33.1 \%)$
$l_{p, 2}$	RS evap. lim	$[-]$	$[10^{-5}, 1.00]$	0.03	$0.06 \; (217.9 \%)$
$K_{s, 2}$	SHC	[mm h$^{-1}$]	$[1.00, 10^4]$	1032.1	$979.5 \; (94.9 \%)$
$\gamma _2$	Flow exponent	$[-]$	$[10^{-5}, 50]$	3.67	$1.31 \; (35.6 \%)$
$S_{i, \max , 2}$	IS height	[mm]	$[10^{-5}, 10.00]$	2.43	$3.65 \; (150.5 \%)$
$k_{v, 2}$	Evap. factor	$[-]$	$[10^{-6}, 10^2]$	3.75	$1.11 \; (29.6 \%)$
$\omega _{f, 2}$	Recharge fract	$[-]$	[0.00, 1.00]	0.003	$0.01 \; (233.3 \%)$
$Q_b$	Base-flow	$[10^3 \, \text {m}^3 s^{-1}]$	[0.00, 0.10]	0.002	$0.01 \; (700.0 \%)$
$\alpha$	Noise decay	[h]	$[10^{-5}, 5 \cdot 10^3]$	30.6	$20.2 \; (66.1 \%)$

Compared to the least-squares calibration results (see “Least-squares calibration”), the overestimation of flow as represented by the $\overline{VCC}$ criterion could be substantially reduced and the *NSE* and *KGE* criteria could be increased with Bayesian inversion (see Table 2). The MLE simulation showed a system behaviour with steeper early recession and a less rapid change towards exponential recession. The MLE simulation is dominated by quick exponential and power-law related behaviour during early recession.

As shown in Figs. 6 and 12, simulation uncertainty is larger during recession periods compared to peak flow estimates. During the first $\approx 2$ months of 2014, flow was systematically underestimated, similarly to the least-squares calibration results. The $5--95$%-quantile range generally covers the data well during the calibration period. However, during late 2014 and early 2015, observed late recessions are not well covered and flow is generally overestimated.

**Fig. 7:**
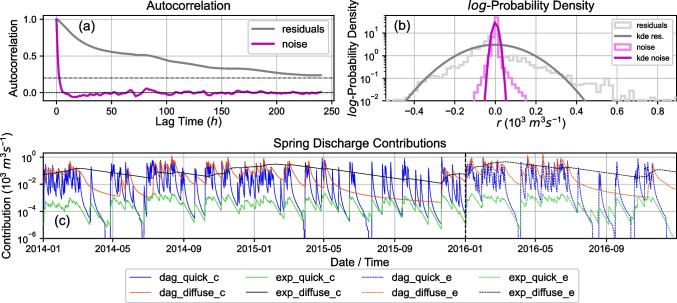
Model diagnostics for data-model-combination 2 (utilizing data from 2014–2015) after Bayesian inversion using the parameter MLE; **a** autocorrelation functions for the residuals and the noise; **b** histograms and Gaussian kernel density estimates (KDE) of the residual and noise distributions; **c** contributions to total spring discharge associated with different response functions, the vertical dashed line separates calibration and testing period; response functions are abbreviated for Dagum (dag) and exponential (exp), subscripts denote calibration (**c**) and evaluation/testing (**e**)

Detailed results of model performance as well as flow contributions from the different flow paths are shown in Fig. 7. From Fig. 7c, the representation of hydrological processes can be observed via the different contributions of the response functions to total spring discharge. It is noted that flow contributions of $Q_m = 0 \cdot 10^3 \, \text {m}^3\,\text {s}^{-1}$ vanish in logarithmic scale and appear as gaps.

The parameters of recharge model 1 (see Table 3) show a recharge regime reacting on shorter time scales compared to recharge model 2, having smaller root zone storage $S_{r, \max }$, higher hydraulic conductivity $K_s$, and larger exponent of non-linearity $\gamma$. The interception storage $S_{i, \max }$ is larger compared to recharge model 2. Root zone evaporation limitation $l_p$ was small for both recharge models but the vegetation factor $k_v$ was found unrealistically large for recharge model 2. The nearly unlimited root zone evaporation effectively reduces the diffuse recharge flux during long dry periods. The small recharge fractionation parameter $\omega$ represents a recharge regime dominated by diffuse recharge.

Similarly, the global flow regime is controlled by diffuse response functions (Fig. 7c). Quick flow components and early recession are dominantly represented by the quick flow Dagum response function, which largely fluctuates on short time scales. Exponential quick flow shows the smallest mean contribution and fluctuates on short time scales as well but not as largely. The diffuse flow Dagum response function contribution represents intermediate flow conditions, reacting on longer time scales. Later recession is dominantly represented by the diffuse flow exponential response function, having the largest mean contribution over the calibration period and reacting slowly to recharge events.

A substantial reduction in residual autocorrelation was apparent when using a noise model (Fig. 7a and b). With that, the assumption of uncorrelated residuals (see section 2.4.2) could be met more accurately. The noise distribution (Fig. 7b) had smaller variance compared to the raw residuals but was still far from a normal distribution as shown by the Gaussian kernel density estimates (KDE).

Especially large parameter standard errors were observed for $A_{q, 1, 2}, \; a_{q, 1, 2}, \; l_{p, 2}, \; S_{i, \max , 2}, \; \omega _{f, 2}$, and *d*. The large uncertainty in $A_{q, 1, 2}, \; a_{q, 1, 2}$ can be explained through the small $\omega _{f, 2}$; the quick flow fraction is so small that those values hardly affect total spring discharge and are hard to infer. Even though $\omega _{f, 2}$ has a large standard error as well, such relative changes from the MLE do not dominate the residual series and therefore the likelihood in Eq. (18). Because no long dry base flow period was apparent in the calibration period, the base flow parameter $Q_b$ is similarly not well informed.

#### Model performance for the testing period

The final simulation for the testing period was performed using the parameter MLE; fit metrics are shown in Tables 4 and 5. It is noted that in Table 4, the overall score is computed as $score = 0.4 \cdot KGE + 0.4 \cdot \overline{VCC} + 0.2 \cdot NSE$. The effort in Table 4 refers to the time needed to develop and calibrate the model (Jeannin et al. 2021); model development took approx. 12 h, least-squares calibration took approx. 30 min for all models combined, and MCMC sampling took approx. 9 h, resulting in an effort of approx. 1 day. Having a total score of 0.90, the TFN model of this study outperformed all other models from the Karst Modelling Challenge. This could be attributed to the good volume conservation performance of the TFN model compared to other models. Volume conservation was especially well met in rising limbs and flood recession (see Table 5), while base flow and undetermined flow were still overestimated. Similar characteristics were observed for the *KGE*, while *NSE* values did not indicate a superior performance. Table 5 suggests that the TFN model performs especially well for dynamic periods, while base flow cannot be represented as accurately.

**Table 4:** Comparison of global evaluation fit metrics of the TFN model to the two best performing models from the Karst Modelling Challenge (Jeannin et al. 2021); the evaluation fit metrics were prepared by the main author of the KMC, P.-Y. Jeannin, for the testing period; see Jeannin et al. (2021) for the results of other studies

Study	Model	$KGE \; [-]$	$\overline{VCC} \; [-]$	$NSE \; [-]$	Effort	Score
This Study	Pastas	0.86	1.03	0.73	1 day	0.90
BRGM France	Gardenia	0.83	0.85	0.83	1 day	0.84
Uni-Freiburg	Varkarst	0.80	0.85	0.79	1 day	0.82

**Table 5:** Comparison of flow-component specific evaluation fit metrics of the TFN model to the two best performing models from the KMC (Jeannin et al. 2021); the evaluation fit metrics were prepared by the main author of the Karst Modelling Challenge, P.-Y. Jeannin, for the testing period; flow components are abbreviated for rising limb (RL), flood recession (FR), base flow (BF), and undetermined flow (UF); see Jeannin et al. (2021) for the detailed results of other studies

Study	$\overline{VCC} \; [-]$				$NSE \; [-]$				$KGE \; [-]$			
	RL	FR	BF	UF	RL	FR	BF	UF	RL	FR	BF	UF
This study	0.92	0.95	1.45	1.22	0.68	0.58	0.52	0.73	0.90	0.93	0.31	0.77
Gardenia	0.89	0.83	0.89	0.85	0.80	0.71	0.80	0.83	0.82	0.82	0.83	0.80
Varkarst	0.95	0.87	0.72	0.73	0.71	0.73	– 0.72	0.51	0.70	0.76	0.48	0.66

**Fig. 8:**
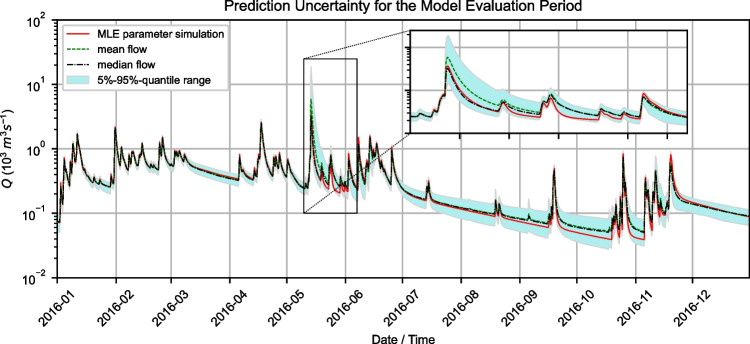
Simulations for data-model-combination 2 (utilizing data from 2014–2015) after Bayesian inversion for the testing period in 2016, showing the simulation mean, median, 5%-95%-quantiles, and the simulations obtained with the parameter MLE

**Fig. 9:**
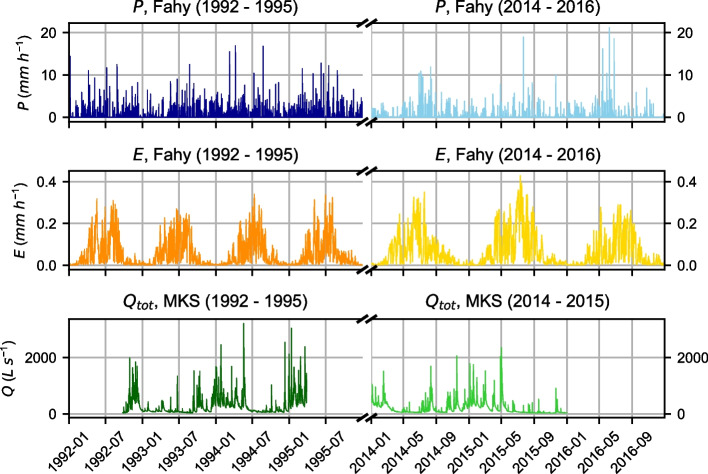
System input and output data used for calibration, Bayesian inversion, and testing

**Fig. 10:**
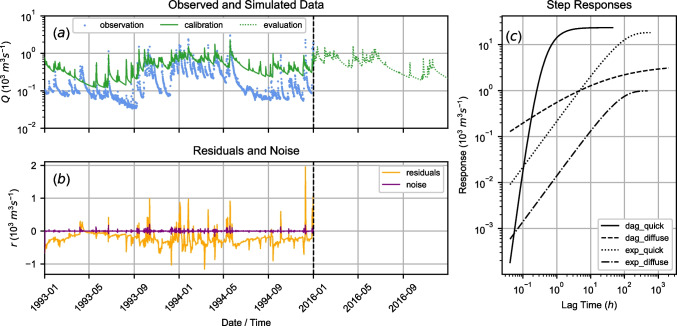
Results for data-model-combination 1 (utilizing data from 1993–1994) after least-squares calibration; response functions are abbreviated for Dagum (dag) and exponential (exp); **a** observed and simulated spring discharge, the vertical dashed line separates calibration and testing period; **b** residuals and noise; **c** calibrated step response functions

**Fig. 11:**
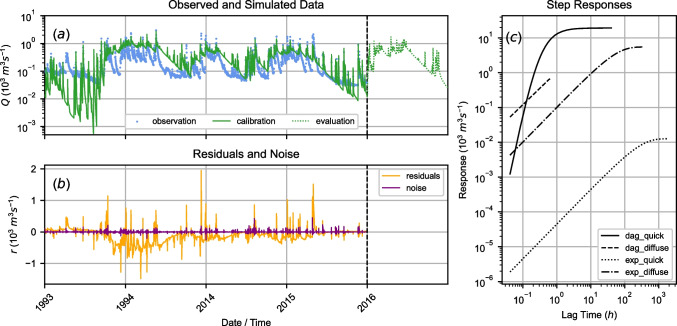
Results for data-model-combination 3 (utilizing data from 1993–1994 and 2014–2015) after least-squares calibration; response functions are abbreviated for Dagum (dag) and exponential (exp); **a** observed and simulated spring discharge, the vertical dashed line separates calibration and testing period; **b** residuals and noise; **c** calibrated step response functions

To incorporate the uncertainty from Bayesian inversion into the analysis of model simulation during the testing period, simulations were carried out for the testing period using 1000 randomly chosen posterior samples. Mean and median spring discharge were computed for every time step as well as a 5–95%-quantile ranges (Fig. 8). Mean and median simulated flow were generally higher compared to the MLE simulation, which was especially evident during recession periods. The width of the uncertainty interval increased with the duration of flood recession. Peak flows were generally estimated similarly for MLE simulation, mean, and median simulated flow. The peak flow event in May 2016 was estimated higher with mean values of flow and lower with median values. Subsequent peak flow events in June 2016 were estimated to be larger by the MLE simulation compared to mean values. Those deviations of the MLE simulation results all lie within the 5–95%-quantile range of simulated flows, and can thus be said to be reliable estimates.

## Discussion

### Calibration and uncertainty quantification

#### Least-squares calibration

It has been shown with least-squares calibration that the choice of the calibration data set affects the (initial) model performance assessment (see “Least-squares calibration”). It has furthermore been shown that the chosen three step optimization scheme for the least-squares calibration was not sufficient to find the (single) parameter set, which best reflects the observations during calibration. This is supported by the improvement of calibration fit metrics show in Table 2. Bayesian inversion with MCMC resulted in a single best parameter set used for final simulation during the testing period, which performed better than least-squares estimates. Furthermore, MCMC sampling provided rigorous estimates of parameter and simulation uncertainty without the limiting assumptions of a linear model or a pre-defined parameter (posterior) distribution type. Particularly in the light of equifinality, a strong argument is made that UQ should always be included in a thorough modelling study involving TFN models (e.g., Gupta et al. 2009). Even though the computational cost for MCMC sampling is magnitudes larger compared to least-squares optimization, the small computational cost of a single model run motivates Bayesian inversion for a single data-model-combination. Computational cost may be reduced by shortening the warmup period but still ensuring a physically viable state of the recharge model prior to the calibration period.

#### Comparison to other modelling approaches from the KMC

Different calibration strategies were followed by the two modelling approaches of the KMC which are compared in detail to the TFN modelling approach in this study (see Tables 4 and 5). While for the Gardenia model (lumped reservoir model), an iterative optimization—similar to least-squares optimization—was used, for the Varkarst model (semi-distributed model), an MCMC algorithm was adopted (see the supplementary materials in Jeannin et al. (2021)). Conceptually, those approaches have different backgrounds and in the present study, a combination of these approaches was used. This suggests that the choice of the specific calibration methodology (i.e., least-squares-type or Bayesian) not necessarily pre-determines performance of a modelling approach during a testing period. It is possible, however, that the performance of the VarKarst and Gardenia models could be improved by implementing the same parameter estimation strategy used in the present study.

The TFN model presented in this study has substantially more parameters compared to the Gardenia ($\textbf{m} = 9$) and Varkarst ($\textbf{m} = 8$) models. Other lumped modelling approaches presented in the karst modeling challenge use a similar or even larger number of parameters compared to the TFN model. Generally, there is no clear correlation between a decreasing number of parameters and increasing model performance. With the present TFN model, a trade-off is being made by introducing more parameters but enabling the model to represent more complex system and recession behaviour, which has been shown to produce good results.

#### Bayesian inversion and uncertainty quantification

**Fig. 12:**
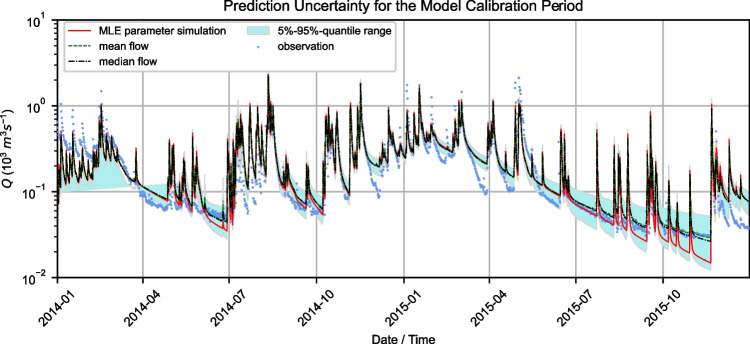
Simulation results for data-model-combination 2 (utilizing data from 2014–2015) after Bayesian inversion for the calibration period, showing the simulation median, 5–95%-quantiles, and the results obtained with the parameter MLE

The resulting MCMC posterior 5–95%-quantile range of flow simulation (Fig. 12) during the calibration period failed to cover several peak flow events and recession periods, which was especially evident from mid 2014 to mid 2015. It was observed in that period that the lower and upper quantile range limits followed a power-law during late recessions, while late recession behaviour in other periods with generally less total spring discharge were exponential. The quantile range limits therefore do not represent the general behavioural limits (e.g., exponential or power-law related recession) of the model in periods with generally more total spring discharge, but are biased towards power-law related behaviour. This was alleviated for periods with generally lower discharge, e.g., from mid 2015 to late 2015, where the upper limit followed an exponential law during late recession and the lower limit followed a power-law recession for longer. Therefore, the dynamic deviation from power-law to exponential recession is critical in representing spring discharge dynamics, which could in theory not be represented with one single instance of the non-linear recharge model. These observations motivate the (implicit) incorporation of structural variability in the TFN model structure with two recharge models, as this source of uncertainty could not have been incorporated in a structurally rigid model.

While the 5–95%-quantile range around the mean and median simulated flow are narrow in the calibration and testing periods, the uncertainty in flow simulation generally increased with the duration of recession periods, indicating uncertainty in the tail of the response functions. This was previously observed in similar studies (e.g., Denić-Jukić and Jukić 2003; Jukić and Denić-Jukić 2006) and can hardly be alleviated in practical applications as all response functions represent aggregated system behaviour and as linearity as well as time invariance is assumed.

The TFN model furthermore suggests a diffuse recharge regime for both recharge models after Bayesian inversion (see “Bayesian model calibration and uncertainty quantification”), which can be supported by field measurements (Perrin et al. 2003; Jeannin et al. 2021). During early recession, power-law related behaviour is dominant and subsequently followed by exponential behaviour during later recession. This representation of hydrological processes is in agreement with the findings of Hergarten and Birk (2007); Birk and Hergarten (2010). In the TFN model output, discharge contributions related to diffuse processes show fluctuations with lower frequency and smaller magnitude compared to their quick-flow counterparts, which agrees with general karst system functioning (see section (Bakalowicz 2005; Hartmann et al. 2014)). Consequently, the TFN model can be said to give a conceptually feasible and realistic system representation. This is furthermore supported by the fact that the base flow parameter $Q_b$ was inferred to be virtually zero (see Table 3), indicating an autogenic recharge regime, which corresponds to field observations (Jeannin et al. 2021).

It has been shown in Fig. 7 that noise modelling greatly reduced residual autocorrelation, better meeting the assumption of independent residuals during least-squares calibration and Bayesian inversion. However, the noise model was not capable of making the residual series normally distributed, although the distribution showed a smaller variance compared to the raw residuals (see Fig. 7). Still, the reduced autocorrelation was assumed to improve model calibration and inversion robustness by better meeting the assumptions of residual independence. Independently of the modelling approach used, noise modelling can always be performed to improve model calibration or inversion robustness and rigor (von Asmuth et al. 2002; von Asmuth and Bierkens 2005) and our results encourage the utilization of noise modelling in future studies.

### Limitations and transferability of TFN models to other systems

From the analysis of model performance in the calibration (see “Least-squares calibration”) and testing periods (see “Bayesian model calibration and uncertainty quantification”) it can be said that the TFN model is applicable to simulate spring discharge of the Milandre karst system, also for previously unseen conditions (testing period).

While model performance was shown to be good according to various criteria, the general lumped approach of TFN modelling naturally comes with corresponding drawbacks. The conduit network structure of the karst system could neither be explicitly represented nor be inferred or characterized with the TFN approach. Approaches exist in that respect, relating karst system structure to recession behaviour (Kovács 2003; Kovács and Sauter 2008; Hergarten and Birk 2007; Birk and Hergarten 2010), which might be incorporated or reflected in the definition of response functions in future studies.

The diffuse recharge regime of the studied karst system results in specific hydrological behaviour and only represents a single set of characteristics from the wide range of karst system characteristics (Bakalowicz 2005; Hartmann et al. 2014; Stevanović 2015). It thus cannot be concluded that the TFN modelling approach used here is applicable to all kinds of karst systems and recharge regimes; further studies for other karst systems are needed to show wide-range and general applicability. For subsequent studies, the large database of Olarinoye et al. (2020) offers a great possibility to test the approach on a number of different karst systems globally.

## Conclusions

It has been shown that the TFN model was able to simulate karst spring discharge well according to multiple fit metrics, outperforming all other models from the Karst Modelling Challenge. Model calibration via Bayesian inversion resulted in a more reliable parameter set compared to least-squares calibration and offered insight into parameter, conceptual, and simulation uncertainty.

Model diagnosis showed that the TFN modelling approach resulted in a realistic representation of the recharge process, where the observed diffuse system behaviour was reflected in parameter values and simulation outcomes.

Early recession periods were dominated by power-law behaviour and medium to late recession periods were more dominantly represented by exponential behaviour, giving an appropriate and conceptually feasible system representation. It was found that the uncertainty in simulated flow generally increased with the duration of long recession periods, indicating uncertainty in the tail of the response functions, which was previously observed from other transfer function model studies.

TFN modelling holds much potential for further development, extending it with, e.g., non-linear behaviour or alternative recharge representations. The great model performance for simulation during previously seen and unseen periods, the physical interpretability, and the low computational cost of the approach ultimately motivate the utilization of TFN modelling in future studies and for other types of karst systems.

## Supplementary information

All data as well as a Jupyter Notebook containing the Python code necessary to run the models is available together with the adapted version of Pastas that supports the two-component non-linear recharge model under https://doi.org/10.5281/zenodo.7715000.

## Data Availability

All data underlying this article are available from the supplementary information in the online version. Under 10.5281/zenodo.7715000 all data and code needed to reproduce all results in the article are available.

## Appendix

See Figs. 9, 10, 11 and 12.

20 $$\begin{aligned} \overline{VCC}&= \frac{1}{N} \sum _{t=0}^N \frac{V_{\text {sim}, t}}{V_{\text {obs}, t}}, \end{aligned}$$

21 $$\begin{aligned} NSE&= 1 - \frac{\sum _{t=0}^N (Q_{\text {sim}, t} - Q_{\text {obs}, t})^2}{\sum _{t=0}^N (Q_{\text {obs}, t} - \overline{Q}_{\text {obs}})^2}, \end{aligned}$$

22 $$\begin{aligned} NSE_{\text {lin}}&= 1 - \frac{\sum _{t=0}^N \mid Q_{\text {sim}, t} - Q_{\text {obs}, t} \mid }{\sum _{t=0}^N \mid Q_{\text {obs}, t} - \overline{Q}_{\text {obs}} \mid }, \end{aligned}$$

23 $$\begin{aligned} KGE&= 1 - \sqrt{(\textbf{r} - 1)^2 + \left( \frac{\sigma _{\text {sim}}}{\sigma _{\text {obs}}} -1 \right) ^2 + \left( \frac{\overline{Q}_{\text {sim}}}{\overline{Q}_{\text {obs}}} - 1 \right) ^2}, \end{aligned}$$

where the subscript *t* denotes the time index, subscripts sim, obs denote simulated and observed values, respectively, $\overline{Q}$ is the mean flow, $\textbf{r}$ is the linear correlation coefficient between observations and simulations and $\sigma$ is the discharge standard deviation. $\overline{VCC}$ is not bounded and can take any value in $(-\infty , \infty )$. *NSE*, $NSE_{\text {lin}}$, and *KGE* can take any value in $(-\infty , 1]$.
